# A Simple, Rapid Method for Determination of Melatonin in Plant Tissues by UPLC Coupled with High Resolution Orbitrap Mass Spectrometry

**DOI:** 10.3389/fpls.2017.00064

**Published:** 2017-01-25

**Authors:** Tiantian Ye, Yan-Hong Hao, Lei Yu, Haitao Shi, Russel J. Reiter, Yu-Qi Feng

**Affiliations:** ^1^Key Laboratory of Analytical Chemistry for Biology and Medicine, Department of Chemistry, Ministry of Education, Wuhan UniversityWuhan, China; ^2^Hainan Key Laboratory for Sustainable Utilization of Tropical Bioresources, College of Agriculture, Hainan UniversityHaikou, China; ^3^Department of Cellular and Structural Biology, The University of Texas Health Science Center, San AntonioTX, USA

**Keywords:** LC-MS, melatonin, Orbitrap, rice, stable isotope

## Abstract

Melatonin (MLT) was involved in regulating various stages of plant growth and development. However, due to the low concentration and complex matrixes of plant, the analysis of MLT is a challenging task. In this study, we developed a rapid and efficient method with simplified sample preparation by employing UPLC coupled with a high resolution Orbitrap mass spectrometry, and stable isotope-labeled MLT (MLT-*d*_4_) was first used as internal standard in the developed analytical method. In the developed method, we used one-step liquid–liquid extraction to purify the crude extracts both from shoot and root of rice for the analysis, which remarkably simplify the sample preparation process. The method exhibits high specificity and recovery yield (>96.4%). Good linearities were obtained for MLT ranging from 0.01 to 20 ng/ mL with determination coefficient (*R*^2^) of 0.9991. The limit of detection for MLT was 0.03 pg. Reproducibility of the method was evaluated by intra-day and inter-day measurements and the results showed that relative standard deviations were less than 7.2%. Moreover, MLT quantification was accomplished by using only 100 mg fresh plant tissues. Additionally, the established method was successfully applied to investigate the spatiotemporal distributions of MLT in rice under cadmium (Cd) stress condition. We found that the content of MLT in shoot and root of rice increased under Cd stress, suggesting that MLT would play a crucial role in modulating the responses to Cd stress in different plant tissues.

## Introduction

Studies on melatonin (*N*-acetyl-5-methoxytryptamine, MLT) in plants have attracted great attention of researchers in recent years. MLT is an indoleamine that has been found to widely distribute in the animal kingdom (Arnao and Hernández-Ruiz, 2015). As an animal hormone, especially a neurohormone (Reiter, 1991), it plays a vital role in vertebrates. Since the first discovery and detection of MLT in plants by Dubbels et al. (1995) and Hattori et al. (1995), successive studies have been invested in its possible physiological functions in plants. In the past 20 years, MLT has been found in more than 140 plant species(Hardeland et al., 2007; Zohar et al., 2011; Gomez et al., 2013; Arnao, 2014; Feng et al., 2014). Moreover, it was reported to be involved in almost all of the plant growth and development stages, from seeds germination to the senescence (Hardeland et al., 2007; Paredes et al., 2009; Arnao and Hernández-Ruiz, 2015; Reiter et al., 2015; Hardeland, 2016). Additionally, many studies have shown that MLT could effectively protect plant tissues from the damage caused by a variety of abiotic and biotic stresses, such as drought stress (Shi et al., 2015b,c), salt stress (Shi et al., 2015b,c), cold stress (Bajwa et al., 2014; Shi et al., 2015b,c), heat stress (Byeon and Back, 2014; Shi et al., 2015d), and pathogen infection (Yin et al., 2013; Shi et al., 2015a).

Accurate and sensitive determination of endogenous MLT enabled us to better understanding its physiological functions, biological synthesis and metabolic pathways, and the regulatory networks. Various quantitative approaches have been developed to analyze MLT content in plants. Immunoassays such as radioimmunoassay (RIA) and enzyme-linked immunosorbent assay (ELISA) have long been applied to MLT quantification (Dubbels et al., 1995; Hattori et al., 1995; Pape and Lüning, 2006; Gomez et al., 2013). ELISA kits are commercially available, along with its ease of operation make it a convenient and common tool for MLT analysis (Pape and Lüning, 2006). However, the cross-reactivity of antibodies is inevitable, which leads to reduced specificity and accuracy (Huang and Mazza, 2011a; Gomez et al., 2013). Gas chromatography coupled to mass spectrometry (GC-MS) has also been used to the qualitative and quantitative analysis of MLT, although it has improved sensitivity and specificity, the requirement for sample volatile derivatization presents a disadvantage for its application to MLT determination (Badria, 2002; Ragab et al., 2006). The sensitivity of capillary electrophoresis (CE) methods has also been reported to be similar to the high performance liquid chromatography (HPLC) approaches (Gomez et al., 2015). HPLC is the most commonly used technique for the analysis of MLT. In this case, different detectors have been used, such as fluorescence detector (FD) (Lavizzari et al., 2006; Pape and Lüning, 2006; Arnao and Hernández-Ruiz, 2007; Padumanonda et al., 2014), electrochemical detector (ECD) (Hernández-Ruiz et al., 2004, 2005; Reiter et al., 2005; Zettersten et al., 2009), and UV (Hosseinian et al., 2008; Ly et al., 2008). However, compared to the MS detector, these detectors exposed their shortcomings of low sensitivity and specificity. LC coupled with MS has been proved as a powerful method for MLT analysis because of its high sensitivity, reproducibility and accuracy (Cao et al., 2006; Hernández-Ruiz and Arnao, 2008; Huang and Mazza, 2011b; Gomez et al., 2012, 2013).

Unfortunately, it is unpractical to directly analyze endogenous MLT in plant tissues by LC-MS due to its low concentration and the complicated matrix of plant extracts. To overcome the difficulty, different sample purification techniques such as liquid–liquid extraction (LLE) (Arnao and Hernández-Ruiz, 2009; Huang and Mazza, 2011a,b; Byeon and Back, 2014), solid phase extraction (SPE) (Huang and Mazza, 2011a; Gomez et al., 2015), microextraction by packed sorbent (MEPS) (Mercolini et al., 2012), HPLC purification (Pape and Lüning, 2006) were commonly employed to enrich MLT from plant tissues. As the classical techniques, LLE and SPE are the most used methods for purification of MLT (Huang and Mazza, 2011a,b; Gomez et al., 2015). At the previous stage, a large volume of solvent and a large amount of plant tissues were needed when LLE was utilized. Thanks to the development of detection techniques, the extraction solvent volume for LLE and the plant amount needed could be decreased, and the LLE merits of fast and easy operation could be kept.

Moreover, matrix effects caused by endogenous interferents often occur during the analysis of low abundant metabolites in the complicated matrix of plant extracts using LC-MS. Nevertheless, these effects can be compensated by using isotope of dilution techniques (Barkawi et al., 2010; Porfírio et al., 2016). Stable isotope-labeled compound(s) is (are) essential internal standard(s) for their physical and chemical similarities with the original analyte(s), providing correction for loss analyte during the experiment procedures and matrix effects during ionization (Barkawi et al., 2010; Porfírio et al., 2016). However, isotope dilution technique has not been reported to be applied to the analysis of MLT content in plants.

Additionally, a highly sensitive and selective MS method was crucial to identify analytes of intrest from complicated matrix of plant. The higher specificity of the technique also leads to a higher sensitivity due to the reduced background noise. Multi reaction monitoring (MRM) (Gomez et al., 2012, 2013; Kocadali et al., 2014) and selective reaction monitoring (SRM) modes (Huang and Mazza, 2011b) have been used previously to the quantification of MLT. And they have been reported to be powerful tools to improve the sensitivity and specificity of analytes. Ions at m/z 216 and 174 were chosen as MS/MS signatures for MLT and have proven particularly useful for quantification (Huang and Mazza, 2011b; Gomez et al., 2012; Gomez et al., 2013; Kocadali et al., 2014). However, the low mass resolution used in these studies could result in false positives, specifically in case of trace metabolites like MLT. Therefore, high-resolution LC-MS is of utmost importance in identification of trace metabolites like MLT or IAA allowing for high sensitivity and appropriate specificity even from crude plant extracts (Yu et al., 2014).

Herein, we developed a rapid, sensitive, and efficient method for the analysis of MLT content by employing a high resolution Orbitrap MS. It enables us to minimize the steps of sample preparation. For both shoot and root of rice, just one-step LLE was added to purify the crude extracts for the analysis. Furthermore, to correct the loss analyte and matrix effects, stable isotope-labeled MLT (MLT-*d*_4_) was added as internal standard to plant samples prior to extraction. Using the developed method, we successfully investigated the dynamic distributions of MLT in response to Cd stress in shoot and root of rice.

## Materials and Methods

### Chemicals and Reagents

Melatonin standard was purchased from Sigma Chemical (St. Louis, MO, USA). Stable isotope-labeled standard, [^2^H_4_] MLT, was purchased from C/D/N Isotopes Inc. (Pointe-Claire, QC, Canada), Chromatography grade acetonitrile (ACN) and methanol (MeOH) were obtained from TEDIA Co. (Fairfield, OH, USA). Ultra-pure water used in the study was purified with Milli-Q system (Milford, MA, USA). Hydrochloric acid (HCl) was purchased from Sinopharm Chemical Reagent (Shanghai, China).

### Plant Materials

Rice (*Oryza sativa* ssp. *japonica* cv. Nipponbare) seeds were germinated and then transplant under hydroponic conditions in Hoagland’s nutrient solution in a growth chamber with 70–80% relative humidity under photoperiods of 16 h light (28°C)/8 h dark (25°C). After 10 days growing under normal conditions, the seedlings were subjected to Cd stress with a series concentration of CdNO_3_ (0, 100, 200, and 500 μM). Shoot and root of rice were harvested after 5, 10, 15 days stresses separately, weighted and then stored at –80°C immediately after freezing in liquid nitrogen.

### Preparation of Plant Samples

Rice samples (100 mg F.W.) were frozen in liquid nitrogen and finely ground, followed by extraction with 1 mL ACN at 4°C for 12 h in dark. [^2^H_4_] MLT (1.0 ng/g) were added to the samples as internal standards (IS). After centrifugation at 12,000 rpm and 4°C for 10 min, the supernatants were sequentially evaporated under mild nitrogen stream. The evaporated samples followed by re-dissolving in 100 μL 0.1 M HCl, and then extracted with ether (1 mL). The ether phase was collected, dried under nitrogen gas and reconstituted in 100 μL H_2_O/MeOH (80/20, v/v) for further analysis.”

### Instruments and Analytical Conditions

Analysis of MLT was performed on an UltiMate 3000 UHPLC System (Thermo-Dionex) equipped with LTQ Orbitrap MS (Thermo-Fisher Scientific, Waltham, MA, USA). The separation of MLT was achieved on an Acquity UPLC BEH phenyl column (2.1 mm × 100 mm, 1.7 μm, Waters) with a flowrate of 0.2 mL/min. The column oven temperature was set at 35°C. Water (solvent A) and acetonitrile (solvent B) were employed as mobile phase. The gradient was started at an initial composition of 95% A and 5% B, 2–20 min, 40% B, then 23 min linear gradient to 90% B, held for 5 min. A return to the initial conditions was accomplished by a 2 min gradient to 95% B, it was held for 10 min, total chromatographic run time was 40 min.

The MS was set to acquire full MS scan in positive ion mode with a mass range of m/z 150–300 at a resolution of 120,000. Ion source conditions were as follows: heater temperature, 300°C; capillary temperature, 350°C; sheath gas flow, 35 arbitrary; auxiliary gas flow, 15 arbitrary; spray voltage, 3.5 kV; S-lens RF level, 60%. Data acquisition and analysis was performed using Xcalibur v3.0.63 (Thermo Fisher Scientific) and SIEVE v2.2 (ThermoFisher Scientific, USA).

### Determination of Electrolyte Leakage (EL)

Electrolyte leakage (EL) was determined according to the previously reported method (Ye et al., 2015). In brief, the initial conductivity was determined after gently shaking at room temperature for 6 h at 150 rpm using a conductivity meter (Leici-DDS-307A, Shanghai, China). The fully releasing conductivity was measured after boiling at 121°C for 20 min using previous samples. The percentage of EL was determined as the ratio of the initial conductivity to fully releasing conductivity.

### Statistical Analysis

The results were shown as means ± standard errors (*n* = 3), SPSS 13.0 software was used for statistical analysis. Analyses of variance (ANOVA) for variables from measurements were used for testing the species and treatment differences according to Duncan’s method. Different letters above the columns in each figure indicate significant differences at *P* < 0.05.

## Results and Discussion

### General Design of Strategy

**FIGURE 1:**
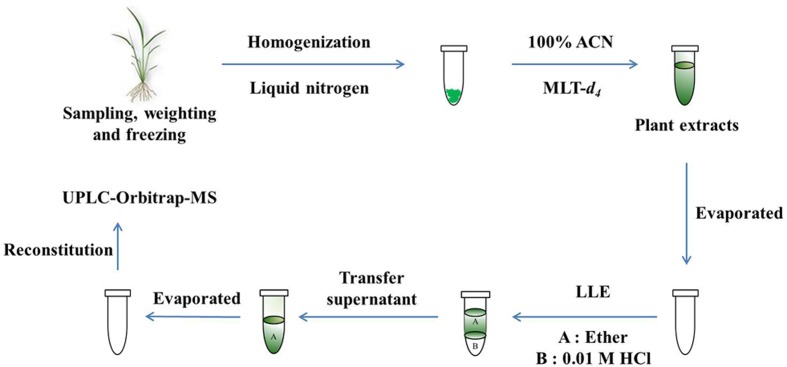
**The general scheme for the analysis of melatonin (MLT) in this study**.

Generally, each additional step would result in loss of analyte to some extent, especially for MLT with the antioxidant capability and light-sensitive characteristic. So, minimizing the number of sample preparation steps is essential. Moreover, high-resolution and accurate MS would benefit for simplifying procedures for sample preparation procedure and reducing analysis time, so an UPLC-high resolution Orbitrap MS was applied to the analysis of MLT in our study. Additionally, stable isotope-labeled MLT (MLT-*d*_4_) was added as internal standard, providing correction for loss analyte during the experiment procedures and matrix effects during ionization. The whole schematic diagram for the analysis of MLT in this study was shown in **Figure 1**.

### Comparison of Different Extraction Solvent

**FIGURE 2:**
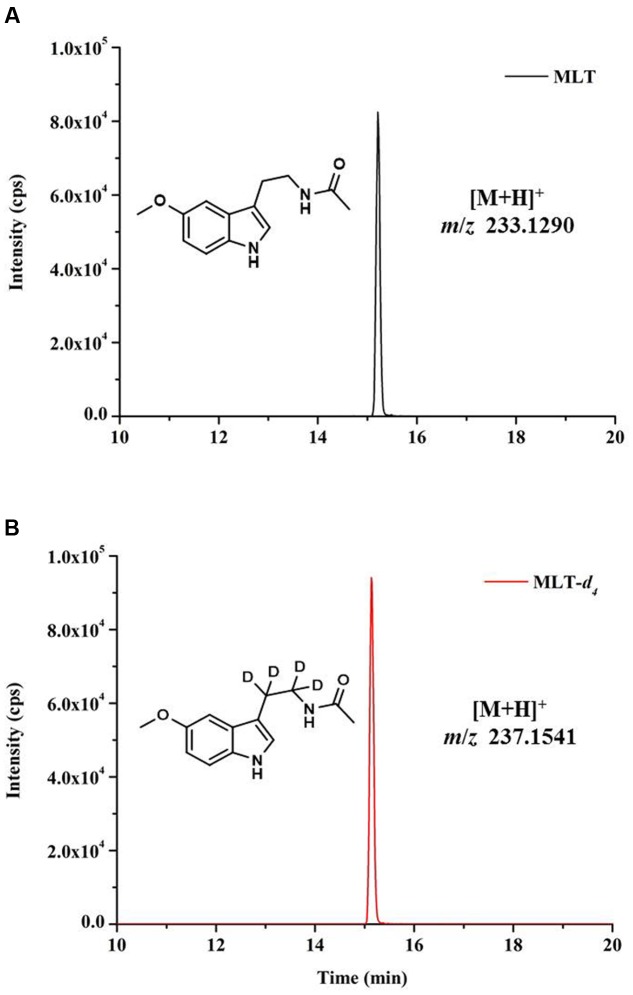
**Chromatograms and accurate mass of MLT standard and MLT-*d*_4_ standard.** MLT standard **(A)** and stable isotope-labeled MLT-*d*_4_ standard **(B)** at the concentration of 10 ng/mL and with injection of 1 μL.

**FIGURE 3:**
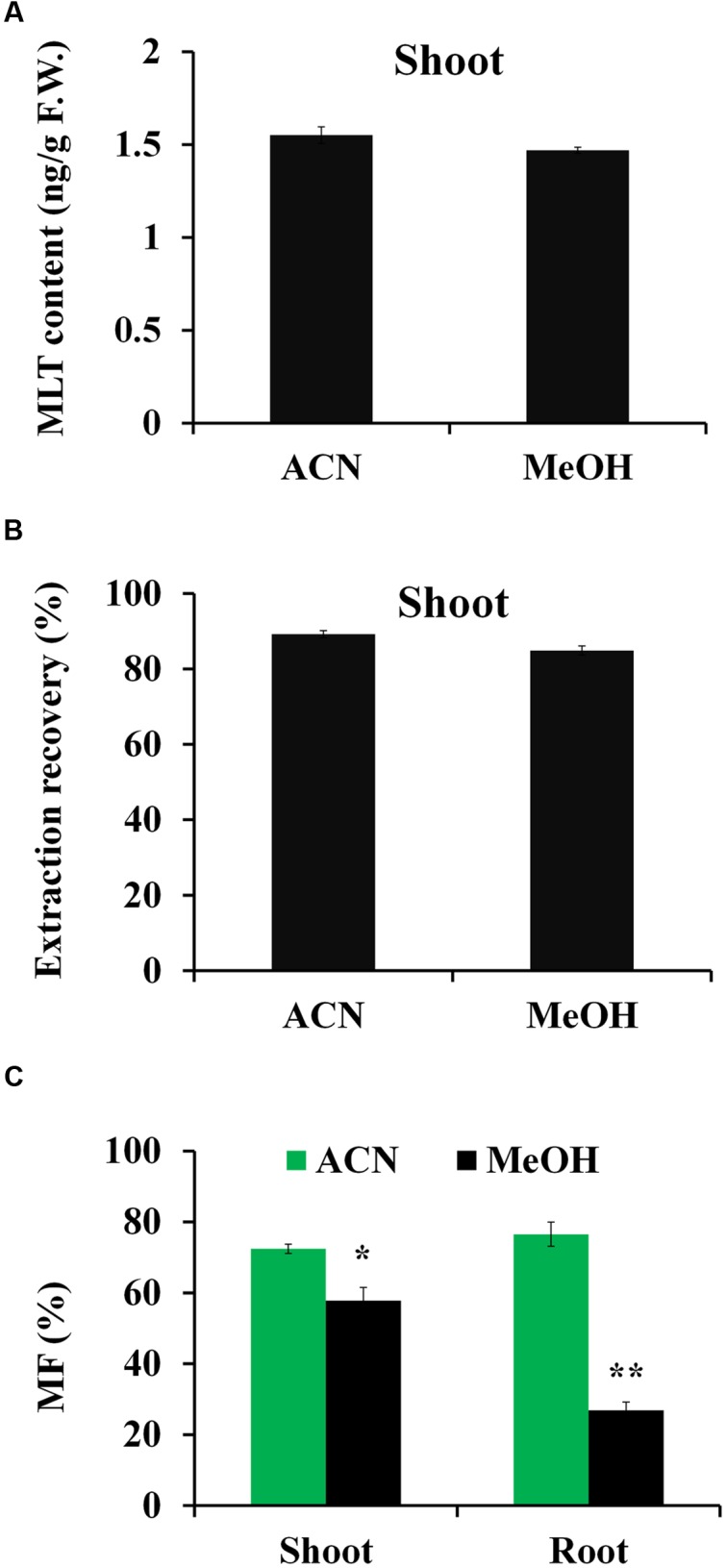
**Comparison of acetonitrile (ACN) and methanol (MeOH) solvent on the extraction efficiencies and matrix effects of MLT.** **(A)** Extraction recoveries of ACN and MeOH solvent for MLT. **(B)** MLT contents of rice shoot using ACN and MeOH as extraction solvent, respectively. **(C)** The matrix effects of ACN extract and MeOH extract for MLT analysis. The calculation of matrix factor (MF): the peak area response for MLT-*d*_4_ in the presence of the plant matrix before injection was compared with peak area response in the absence of matrix. Asterisk symbols ^∗^*P* < 0.05 and ^∗∗^*P* < 0.01 (*t*-test).

Melatonin is amphipathic substances, with antioxidant capabilities and poor ionization efficiency (Huang and Mazza, 2011a). And concentration of MLT in complicated plant matrix is fairly low, whereas numerous of other substances with far greater amounts are present. Therefore, it is important to choose an extraction method that yields good recovery. To achieve high extraction efficiency, two frequently used organic solvents, ACN and MeOH were chosen for the optimization of MLT extraction from plant tissues. The retention time and accurate mass of MLT standard and MLT-*d*_4_ standard were shown in **Figure 2**. Our result showed the extraction efficiencies of MLT by ACN and MeOH were similar, and there is no significant different of MLT content in shoot (MLT signal was severely suppressed in crude extract of root in both solvents, and it will be discussed in next section) of rice using ACN (1.55 ng/g) or MeOH (1.47 ng/g), and the both solvents exhibited good extraction recoveries (>80%) (**Figure 3**).

In plant samples, matrix often play negative roles in MS signals. Due to the complicated matrix of plant extract, the abundant interferents from the plant extract might depress the MS response of MLT. So, the matrix effects were further assessed between ACN extract and MeOH extract. The MS response (peak area) for MLT-*d*_4_ in the presence of the plant matrix was compared to peak area in the absence of matrix to calculate matrix factor (MF) according to Yu et al. (2016). For shoot samples, MF from ACN extract was slightly higher than MeOH extract. But for root sample, a strong matrix effect was observed for MeOH extract (MF, 30%), while approximately more than two times lower than ACN extract (MF, 76%) (**Figure 3**). Therefore, ACN was selected as appropriate solvent for extracting MLT from rice tissues.

### Optimization of Sample Preparation Procedure

**FIGURE 4:**
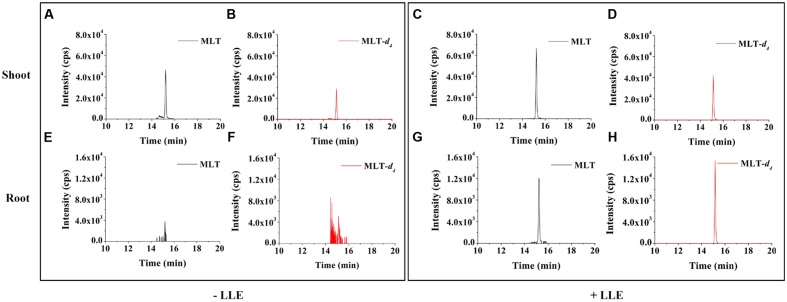
**Comparison of chromatograms with or without LLE purification for MLT analysis.** Chromatograms of MLT standard and MLT-*d*_4_ standard in an extract of 100 mg rice shoot **(A–D)** and root **(E–H)** samples with or without purification of LLE.

In the previous section, we mentioned that MLT signal was strongly suppressed in crude extract of root. To solve this problem, further purification step was needed. Taking advantage of the analyte solubility differences in different solvents, LLE is an effective way to purify the MLT in plants (Chen et al., 2012). Furthermore, LLE can improve the ionization efficiency by decreasing the influence of impurities (Chen et al., 2012). The evaporated sample followed by re-dissolving in 100 μL 0.1 M HCl, and then extracted with ether (1 mL). The results showed that with the purification of LLE, the signal intensity of MLT in root samples was significantly increased, while the intensity of MLT was also increased slightly in shoot samples (**Figure 4**). Therefore, LLE was adopted to eliminate the polar impurities and improve the ionization efficiency of MLT in rice.

### Method Validation

To evaluate the quantitative aspect of the developed method, calibration curve of MLT was established. The MLT standard (0.01–20 ng/mL) was spiked in shoot and root samples (100 mg F.W.). The internal standard MLT-*d*_4_ was added to the sample prior to extraction step. The calibration curves were plotted by the peak area ratio (MLT labeled with MLT-*d*_4_) against the MLT concentrations. As shown in **Table 1**, good linearity was obtained for MLT with coefficient of determination (*R^2^*) of 0.9991. The LODs and the LOQs were calculated as the concentration of the analyte at a Signal/Noise (S/N) ratio of 3 and 10, respectively. The results showed that LOD and LOQ for MLT were 0.03 and 0.11 pg, respectively.

**Table 1:** Calibration characteristics of melatonin (MLT) standard spiked in solution of rice seedling samples.

Analyte	Linear range (ng/mL)	Regression data			LOD (pg)	LOQ (pg)
				
		Slope	Intercept	*R*^2^ value		
Melatonin	0.01–20	0.9538	0.0566	0.9991	0.03	0.11


The accuracy and precision of the developed method were further assessed, respectively, by the recoveries and by the relative standard deviations (RSDs) of intra- and inter-day. Both recoveries and intra- and inter-day RSDs were calculated with MLT standard spiked in rice samples at three different concentrations. The intra-day variation was evaluated by repeating the process for five times within one day and the inter-day variation was investigated on five successive days. For each concentration, five replicated measurements were performed in both shoot and root samples. The relative recoveries were calculated by comparing the peak area ratios of MLT from the spiked rice samples to those from the standard solutions. As shown in **Table 2**, good precisions were obtained with RSD values below 7.2%, and the relative recoveries were in the range of 96.4 to 105.1%, indicating the good reproducibility and accuracy of developed method.

**Table 2:** Accuracy and precision (intra- and inter-day) for the determination of MLT in shoot and root tissues of rice (100 mg F.W.).

Analyte	Tissues	Intra-day precision (RSD, %, *n* = 5)			Inter-day precision (RSD, %, *n* = 5)			Recovery (%, n = 5)		
				
		Low (1 ng/mL)	Medium (5 ng/mL)	High (20 ng/mL)	Low (1 ng/mL)	Medium (5 ng/mL)	High (20 ng/mL)	Low (1 ng/mL)	Medium (5 ng/mL)	High (20 ng/mL)
Melatonin	Shoot	3.76	3.13	2.89	1.28	0.47	1.11	101.35 ± 3.97	96.47 ± 4.07	96.42 ± 1.68
	Root	1.97	6.15	7.23	3.17	5.75	4.71	98.09 ± 6.15	97.33 ± 6.33	105.15 ± 2.98


Furthermore, to assess the general utility of proposed method for determination of MLT in other plant species or different tissues of plant, the method was used to detect MLT in *whole seedlings and leaves of Arabidopsis* (Col-0), rice leaves (*Oryza sativa* ssp. *japonica* cv. Nipponbare), cotton leaves and fibers (*Gossypium hirsutum*), rape leaves (*Brassica napus* L. cv. Zhongshuang 11). As shown in **Table 3**, the content of MLT varies in different plant species and tissues. The MLT content in leaves of one-month-old *Arabidopsis* and *Brassica napus* was similar. In rice leaves (10 days after transplant under hydroponic conditions), the MLT reached 0.58 ng/g F.W., which was about threefolds higher than in *Arabidopsis* leaves. The MLT content in *Gossypium* sp of maturity date was much higher than rice. The relative recoveries from different plant tissues were found to be in the range of 84.3 to 104.2%, indicating the universal applicability of the developed method.

**Table 3:** General utility assessment of proposed method for determination of MLT in different plant species and tissues.

Analyte	Plant species	Plant tissues	Content (ng/g F.W.)	Recovery (%, *n* = 3)
Melatonin	*Arabidopsis*	Whole seedling	0.22 ± 0.01	88.84 ± 5.42
	*Gossypium* sp.	Fibers	0.34 ± 0.01	99.11 ± 9.76
	*Arabidopsis*	Leaf	0.19 ± 0.03	104.21 ± 5.98
	*Brassica campestris* L.	Leaf	0.23 ± 0.02	91.88 ± 3.59
	*Gossypium* sp.	Leaf	0.74 ± 0.01	102.89 ± 2.01
	*Oryza sativa*	Leaf	0.58 ± 0.01	84.28 ± 7.27


Compared with previous LC-MS methods, our proposed method showed the following improvements. Firstly, high resolution MS was used for the detection of MLT. Accurate m/z (5 ppm) of the target analytes was moniterd with high selectivity. The high specificity of the m/z value lead to the decreased matrix interference and improved sensitivity of analytes by reducing the background noise. Secondly, we employed a simple and effective LLE procedure to purify MLT in plant tissues, and the matrix interference got further decreased. Thirdly, the stable isotope dilution strategy was first used for absolute quantification of MLT in plant tissues, and worked well to correct the loss of analyte during the sample pretreatment procedures and signal variation in MS detection.

### MLT Distribution under Cd Stress in Shoot and Root of Rice

Cadmium is one of most toxic pollutions in the present. Unlike the other heavy metal pollutions, Cd is a non-essential nutrient, and seriously affects plant growth and development. Recently, Cd has been reported to regulate MLT content in rice leaves by coordinating the synthesis and degradation genes of MLT (Byeon et al., 2015). And then Hasan et al. found, exogenous application of MLT could mitigate phytotoxicity induced by Cd stress in *Solanum lycopersicum* L., by modulation of phytochelatins biosynthesis, vacuolar sequestration, and antioxidant potential (Hasan et al., 2015). However, our knowledge of MLT for regulating response of plant to Cd stress is still fragmentary (Arnao and Hernández-Ruiz, 2014).

**FIGURE 5:**
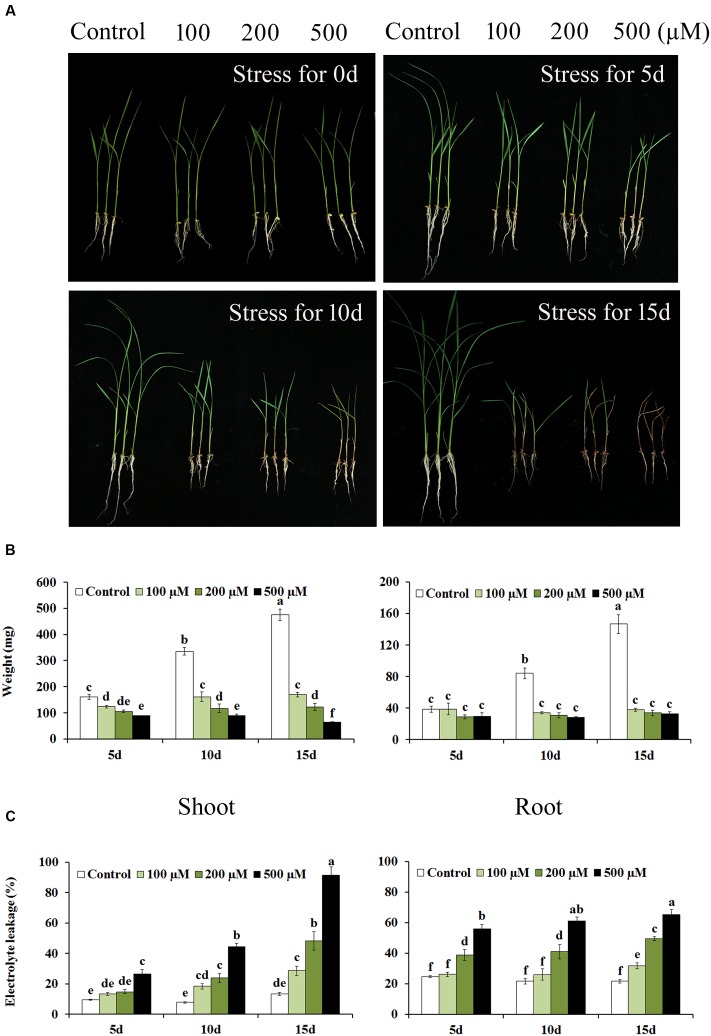
**Comparison of physiological responses to cadmium (Cd) stress in shoot and root of rice. phenotypes** **(A)**, weights **(B)**, EL **(C)** of rice shoot and root under control and stressed condition at designated time intervals. The data represent the means of three independent experiment ± SE, and data followed by different letters are significantly different from each other at *P <* 0.05 according to Duncan’s method.

**FIGURE 6:**
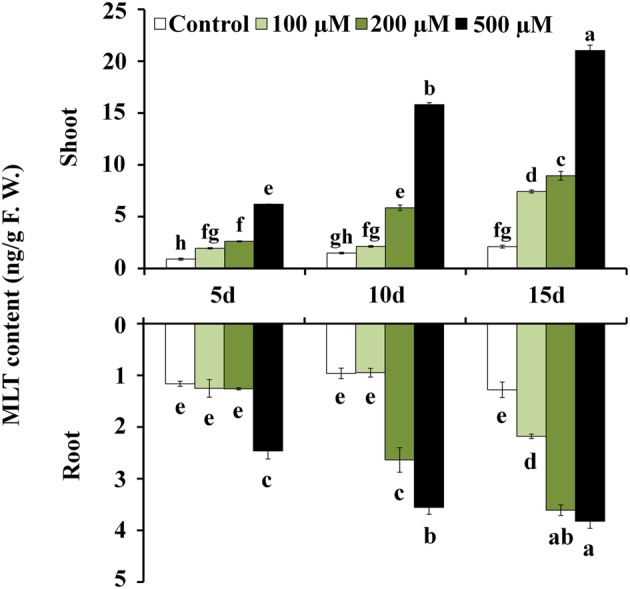
**Changes of MLT level in shoot and root of rice after Cd treatment.** The results shown are means ± SE (*n* = 3), and the results followed by different letters are significantly different from each other at *P* < 0.05 according to Duncan’s method.

In this study, we investigate the distributions of MLT both in shoot and root of rice under Cd stress condition using the established method. 10-day-old rice seedlings were subjected to Cd stress with a series concentration of CdNO_3_ (0, 100, 200, and 500 μM). When exposed to 15 day treatments, significant differences in the growth of rice were observed, suggesting that Cd stress caused serious damages (growth inhibition, wilted leaves) on rice (**Figure 5**). The shoot weight only reached 63.5 mg at 15 day under Cd condition of 500 μM, which were only 13% of control seedlings, while the growth of root was almost completely inhibited with the Cd treatment of 200 and 500 μM (**Figure 5**). Additionally, Cd stress progressively increased the EL with the duration of the treatment. The EL reached 91% at 15 day under Cd treatment of 500 μM (**Figure 5**). These result indicated Cd stress caused severe cell membrane damages and severely inhibited rice growth. Nevertheless, the MLT content in rice shoot was increased with the increasing of Cd concentration and the stress time. After 15 day treatment of 500 μM Cd, the MLT content in rice shoot was up to 21.0 ng/g, which was about 10-folds higher than control (**Figure 6**). Additionally, the MLT levels in rice root under Cd stress were detected as well (**Figure 6**). The results showed that MLT content was also gradually increased over time after Cd treatment in root. Notably, higher concentration of Cd treatment induced higher level of MLT. Under Cd condition of 500 μM at 15 day, the MLT content in root was up to 3.9 ng/g, which was over three times higher than control. Our results suggested that MLT might play a critical role in rice tolerance to Cd stress both in shoot and root tissues. The MLT content was also induced by Cd treatment in root, indicating MLT might be involved in preventing translocation of Cd from root to shoot, and might modulate the responses to Cd stress through the different mechanisms in shoot and root. Further studies are still needed to provide the evidence in support of the involvement of melatonin in Cd tolerance and detoxification, and to illuminate signaling pathway in different tissues of plants.

## Conclusion

In this study, we developed a rapid, sensitive and efficient sample preparation method to accurate measure MLT in crude plants extracts down to 100 mg F.W. by employing a high resolution Orbitrap MS and using stable isotope-labeled MLT (MLT-*d*_4_) as internal standard. Using this approach, we successfully minimize sample purification process into one-step LLE procedure to purify the crude extracts in rice, which greatly simplified the sample preparation procedure and improved the analytical throughput. The established method was successfully applied to investigate the dynamic distributions of MLT responses to Cd stress in shoot and root of rice. The developed method may facilitate to better understanding the physiological functions and regulatory mechanism of MLT in plant.

## Author Contributions

TY designed and performed the experiments, analyzed the data, and wrote the manuscript; Y-HH performed the experiments and analyzed the data; LY performed the experiments; HS and RR revised the manuscript; Y-QF designed the experiments and revised the manuscript; and all authors approved the manuscript.

## Conflict of Interest Statement

The authors declare that the research was conducted in the absence of any commercial or financial relationships that could be construed as a potential conflict of interest.
